# Usage and perceptions of anabolic-androgenic steroids among male fitness centre attendees in Kuwait - a cross-sectional study

**DOI:** 10.1186/s13011-015-0030-5

**Published:** 2015-08-22

**Authors:** Ibrahim Alsaeed, Jarrah R. Alabkal

**Affiliations:** Arabian Gulf University, College of Medicine and Medical Sciences, Manama, Kingdom of Bahrain

**Keywords:** Sport medicine, Anabolic androgenic steroids, Substance abuse, Public health

## Abstract

**Background:**

Considering the recent popularity of bodybuilding and the apparent spread of anabolic androgenic steroid (AAS) use amongst bodybuilding enthusiasts in Kuwait, there is a relative lack of scientific investigation into the use, knowledge and attitudes towards AAS amongst the population at risk of abusing it. Therefore, this study aims to investigate the frequency, knowledge, attitudes and practice of AAS use amongst male fitness centre attendees in Kuwait.

**Methods:**

A cross sectional survey utilizing a self-administered questionnaire was used. Information on demographics as well as knowledge and attitude about and towards the use of AAS was included in the questionnaire. Ten fitness centres in Kuwait were randomly selected and questionnaires were distributed to all individuals leaving each centre on randomly selected days and periods of time for each centre. Overall *n* = 400 questionnaires were distributed.

**Results:**

A total of *n* = 194 questionnaires were returned completed (~49 %). Of the responders, 22.7 % used AAS. The 19–25 age group had the highest occurrence (46.8 %) of first-time AAS use. In contrast with non-users, most (70.5 %) of AAS users believed that having an optimally muscular body can only be achieved by using AAS, and a small minority (6.8 %) believed that AAS usage would have significant harms to health. Only 18.2 % of AAS users had appropriate knowledge regarding the side effects of AAS. Non-users were as much uninformed as AAS users regarding the side effects of AAS.

**Conclusion:**

The usage of AAS is high amongst male gym users in Kuwait and is likely to present an additional burden to the health service. An effective initiative to minimize the burden of AAS abuse should focus on changing the attitudes towards AAS rather than spreading awareness of their side effects.

## Background

Anabolic androgenic steroids (AAS) are synthetic derivatives of testosterone having pronounced anabolic properties and relatively weak androgenic properties [2]. They are used clinically for the treatment of conditions characterized by abnormally low production of testosterone, or muscle wasting [4]. However, since the 1950s, AAS have been used by professional body-builders and increasingly by young adults to improve physical appearance [4, 20]. These non-medical uses of AAS are associated with significant health risks such as cardiovascular, hepatic, endocrine, psychosocial and psychiatric disorders as well as death [8, 19].

Recently it has been reported that approximately 20 % of athletes in the United States use AAS [7]. Life-time prevalence of AAS across western countries is estimated at between 1-6 % with the majority of users being male [3]. Furthermore, the prevalence is estimated at ~38 % amongst gym athletes [18]. Studies on prevalence rate in the Middle East and North Africa (MENA) region are few, and the prevalence rate is estimated at ~22 % amongst gym users in the United Arab Emirates [1], and ~13 % of Iranian youth training as body builders [12]. As regards practice habits, a recent internet-based survey has reported that the majority of users self-administer injectable AAS and reported subjective side-effects for AAS usage (99.2 %); furthermore, 25 % of users are taking AAS, growth hormone and insulin concurrently for anabolic effects [13]. A study of Canadian college students showed that AAS users were taking a variety of substance concurrently to enhance performance including caffeine, pain killers, stimulants and beta-blockers [9].

Several studies have been conducted to assess the knowledge and attitude of people exercising in fitness centers toward the use of AAS. A survey of ~5,000 men and women in Denmark reported that AAS users have more positive attitudes towards AAS use compared to non-users across a variety of sports [15]. In Sweden, a survey of ~4,000 male adolescents showed that fewer users believed AAS to be harmful, while more believed that females preferred males with bigger muscles. Moreover, it showed that AAS users trained more often at gyms, drank more alcohol and used narcotic drugs more often than other male adolescents [11]. A survey of ~150 gym users in the United Arab Emirates reported that 7 % of non-users were planning future use of AAS [1]. In a recent study in Kuwait, the prevalence of AAS users amongst gym members was 11.8 %, and all of them started using AAS when they were less than 20 years old [10].

Across the MENA region, the general public lack information regarding the knowledge and attitudes of AAS users. Considering the recent popularity of bodybuilding and the apparent spread of Anabolic Androgenic Steroid (AAS) use amongst bodybuilding enthusiasts in Kuwait, there is relatively not enough scientific investigation into the use, knowledge and attitudes towards AAS amongst the population at risk of abusing it (namely; fitness centre’s attendees). The aim of this study is to investigate the knowledge, attitudes and practice of AAS use amongst male fitness centres attendees in Kuwait.

## Methods

### Participants

This was a cross-sectional survey of males attending fitness centres in Kuwait. These fitness centres were randomly selected from the national telephone directory. Self-administered questionnaires were then distributed to all individuals leaving each centre on a randomly selected day and period of time which was different for each centre. Overall *n* = 400 questionnaires were distributed. A total of *n* = 194 were returned completed (~49 %).

### Measures/instruments

The survey questionnaire content and structure was broadly based on that of [1], and included a sections on demographics, as well as questions related to knowledge and, attitudes about and towards use of AAS. Briefly, the questionnaire consisted of 34 questions which were distributed as follows: demographics *n* = 3, AAS usage *n* = 4, knowledge and attitude towards AAS *n* = 12, AAS practice habits *n* = 5, healthy lifestyle *n* = 7, and miscellaneous *n* = 3. Questions were closed type and answer choices were a combination of Likert scale and tick-box types. When a questionnaire was returned, it was immediately checked for missing answers by the investigator and, if necessary, the participant was invited to complete them accordingly. One question had a list of 11 undesirable effects, some associated with AAS use and some unrelated to AAS, which was used to test the knowledge about the side effects of AAS. The question read, “adverse effects that can result from AAS use include:” and the responder chose true, false, or don’t know for each of the listed effects. Responders that gave wrong responses or chose “don’t know” for more than seven items in the list were assigned as having ‘inadequate knowledge’ about the potential harms. Responders who gave correct answers for more than seven items in the list were assigned as having ‘appropriate knowledge’. The rest were assigned as having ‘some knowledge’.

### Procedure

Ethical approval was obtained from the Ethics Committee of Arabian Gulf University, Kingdom of Bahrain. Participation in the study was voluntary and all participants signed a consent form prior to participating in the study. Responses were confidential.

### Statistical analysis

Statistical analysis was conducted using SPSS package (version PASW Statistic 18.0.3). Chi-square test was used to test for significant differences.

In our analysis, responders who’ve reported they hadn’t used AAS (non-users) were divided into three groups based on their intentions to use AAS in the future as follows; 1) non-users who’ve reported they had intentions to use AAS in the future (non-users with intentions to use), 2) non-users who’ve reported they may or may not use AAS in the future (non-users with undetermined intentions), 3) non-users who’ve reported having no intention to use AAS in the future (non-users with no intentions).

## Results

### Usage and demographics

**Fig. 1:**
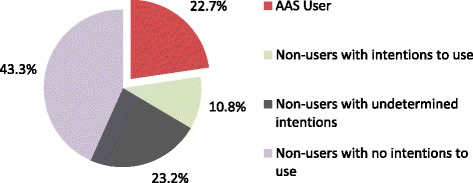
Distribution of participants based on history and intention of using Anabolic Androgenic Steroids

The age distribution of the sample when divided into users and non-users is shown in Table 1. There was a statistically significant difference in age distribution between users and non-users in our sample (*Χ*^2^ = 7.941, df = 4, *p* < 0.05). The percentage of people who’ve used AAS (“AAS users”) in our sample was 22.7 % (Fig. 1). In this sample of responders, the age group at which AAS were first used was 14–18 years (27.7 %); 19–25 years (46.8 %); 26–34 years (17 %); >35 years (6.4 %). In our sample, 4.6 % of participants reported having primary school education level, 23.7 % high school level of education, 22.2 % Associate’s degree, and 49.5 % Bachelor degree or higher. There was no statistically significant difference in level of education between users and non-users of AAS (*Χ*^2^ = 1.963, df = 3, *p* = 0.58).

**Table 1:** Age distribution of users and non-users of Anabolic Androgenic Steroids

Age (years)	14 – 19	20 – 29	30 – 40	>40	Total (number)
AAS users	9 % (4)	56.8 % (25)	25 % (11)	9 % (4)	44
Non-users	22 % (33)	59.3 % (89)	16 % (24)	2.6 % (4)	150
Total	19 % (37)	58.8 % (114)	18 % (35)	4 % (8)	194

Of the responders, 10.8 % were non-users with intentions to use, 43.3 % were non-users with no intentions to use, and 23.2 % were non-users with undetermined intentions (Fig. 1).

### Attitudes to AAS

A high percentage of users (70.5 %) and non-users with intentions to use (67 %) agreed with the statement that having an optimally muscular body can only be achieved by using AAS; the difference between the two group was not statistically significant (*Χ*^2^ = 0.61, df = 2, *p* = 0.737). In contrast, a minority (19 %) of non-users with no intentions to use agreed with that statement; which was significantly less than AAS users (*Χ*^2^ = 35.014, df = 2, *p* < 0.0001) as well as non-users with intentions to use (*Χ*^2^ = 19.451, df = 2, *p* < 0.0001). Non-users with undetermined intentions were much more divided in their attitudes regarding the beneficial effects of AAS on muscular definition (Table 2).

**Table 2:** Differences amongst participants based on history and intention of using Anabolic Androgenic Steroids

Variable	AAS users	Non-user		
		With intentions to use	With undetermined intentions	With no intentions to use
Having an optimally muscular body can only be achieved by using AAS				
True	70.5 %	67 %	34 %	19 %
False	25 %	24 %	36 %	51 %
I don’t know	4.5 %	9 %	30 %	30 %
Do you personally know an AAS user?				
Yes	99.78 %	90.5 %	80 %	64.3 %
Is AAS harmful?				
Yes, very harmful	6.8 %	9.5 %	20 %	50 %
Not very harmful	29.5 %	42.9 %	33.3 %	14.3 %
No, not if it was used correctly	56.8 %	42.9 %	24.4 %	19 %
I don’t know	6.8 %	4.8 %	22.2 %	16.7 %
Use many (>3) supplements and aids to achieve his goals in the gym (other than AAS)				
Does	80 %	28.6 %	24.4 %	10.7 %

A small minority of AAS users (6.8 %) thought that AAS usage is very harmful, 29.5 % thought that it is not very harmful, and 56.8 % thought it is not harmful to health if used correctly. Non-users with intentions to use had the same distribution of attitude towards the harmfulness of AAS (*Χ*^2^ = 1.507, df = 3, *p* = 0.68). In contrast, 50 % of non-users with no intentions to use reported that AAS usage is very harmful, 14.3 % reported it is not very harmful, and 19 % reported it is not harmful if used correctly; this distribution is significantly different from that of the aforementioned two groups of participants (*Χ*^2^ = 33.727, df = 3, *p* < 0.000001, *Χ*^2^ = 19.092, df = 3, *p* < 0.001, respectively) (Table 2).

### Peer effect

Nearly all AAS users (97.8 %) and most non-users with intentions to use (90.5 %) personally knew someone who was using AAS, with no statistically significant difference in the percentage between the two groups (*Χ*^2^ = 1.698, df = 1, *p* = 0.19). The percentage, however, was significantly less (80 %) amongst non-users with undetermined intentions compared to AAS users (*Χ*^2^ = 7.01, df = 1, *p* < .01). On the other hand, only 64.3 % of non-users with no intentions to use knew an AAS user, which is significantly less than the percentage amongst AAS users (*Χ*^2^ = 17.595, df = 1, *p* <0 .0001) (Table 2).

### Reported reasons for not using AAS

Several reasons for not using AAS were reported. When analyzed, being harmful to health was the most important single reason for not using AAS, and having not felt the need to use AAS was the second. Amongst non-users with no intentions to use (*n* = 84); only 58.3 % reported that being harmful to health was their primary reason for not using AAS, and 27.4 % reported that it was not their reason at all. Amongst the non-users with no intentions to use and non-users with undetermined intentions combined (*n* = 129), only 34.1 % reported that being harmful to health is their primary reason for not using AAS, and 49.4 % reported it was not their reason at all.

### Motivation to exercise

A total of 81.8 % of AAS users and 71.4 % of non-users with intentions to use reported that getting muscular body appearance was their primary reason for attending the fitness centre. Amongst non-users with no intentions to use, 39.3 % reported that getting muscular body appearance was their primary reason for attending the fitness centre, which was significantly different from AAS users (*Χ*^2^ = 21.022, df = 1, *p* <0.0001).

### Practice of AAS use

**Fig. 2:**
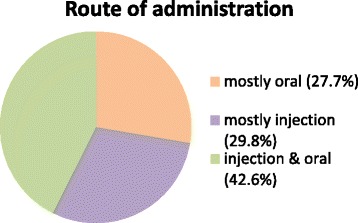
Route of administration of Anabolic Androgenic Steroids

Both oral and injectable forms of AAS were used in approximately equal frequency, and a combination of oral and injectable AAS was the most common practice (42.6 %) (Fig. 2). Furthermore, 53.2 % administered one or more courses of AAS consisting of a combination of ≥ 2 substances.

### Usage of other substances

The frequency of intake of many (≥3) other ergogenic aids and supplements (e.g. growth hormone, Nitric Oxide-containing supplements, creatine-containing supplements, etc.) was highest amongst AAS users (80 %), and was significantly higher than amongst non-users with intentions to use (28.6 %) as well as the prevalence amongst all non-users (17.3 %) (*Χ*^2^ = 15.859, df = 1 *p* < 0.0001, *Χ*^2^ = 61.082, df = 1 *p* <0.0001, respectively) (Table 2). The frequency of smoking was significantly higher (70.5 %) in AAS users than (48.7 %) in non-users (*Χ*^2^ = 6.296, df = 1 *p* < 0.05).

### Source of AAS

Users obtained AAS from a variety of sources as follows: gym coach (62.1 %), individual supplier (58.8 %), whilst travelling abroad (34.6 %), pharmacy or physician (30.8 %), internet order (25.9 %) and friends (24 %).

### Knowledge

Fifty nine percent of AAS users and 24.7 % of non-users reported that they believed they have enough information regarding AAS. When the knowledge about potential adverse effects of AAS was examined, we found that 50 % of users had inadequate knowledge and only 18.2 % of them had appropriate knowledge. There was no significant difference between users and non-users in knowledge of side effects (*Χ*^2^ = 0.561, df = 2, *p* = 0.75).

## Discussion

This study investigated the frequency, knowledge, attitudes and practice of AAS use in Kuwait amongst male fitness centre attendees. The frequency of AAS users was 22.7 %, which is higher than what was reported in a similar study in Kuwait (11.8 %) [10] and what was previously reported amongst Iranian youth training as body builders (~13 %) [12], but less than previously reported for gym athletes (~38 %) [18], and similar to that reported amongst gym users in the United Arab Emirates (~22 %) [1]. Highest frequency of first time AAS usage was in the age group 19–25 years (46.8 %). This finding is somewhat similar to that reported by others in the MENA region [1, 10, 17]; however, in Sweden age of first usage is lower (16–17 years) [16].

Considering that AAS users were as educated as non-users, it does not appear that the level of education was a factor in the decision to use AAS in this study. Both the belief that AAS are crucial to optimum muscular bulk, and personally knowing someone who uses AAS, were associated with AAS use. AAS users and non-users with intentions to use believed more strongly in the benefits of AAS and were less worried about the risks associated with their use. Overall, users perceived that the benefits to muscle bulk outweighed the risks of negative side-effects. These findings are in broad agreement with those reported in other studies of gym users and athletes [1, 10, 14, 15].

It is interesting that only 34.1 % of non-users (excluding non-user with intentions to use) reported that being harmful to health was their primary reason for not using AAS. Most of non-users with no intentions to use AAS have joined the gym for reasons other than body-building, and most of them reported they have not felt the need to use AAS to achieve their desired goal in the gym. This indicate that the main reason gym members did not use AAS was that they didn’t think or feel they needed them, and not because of their side effects. This piece of information could be crucial for any effort toward controlling the spread of AAS abuse.

The use of AAS was linked with the use of other subsances; AAS usage was clearly associated with a much higher frequency of intake of many (≥3) ergogenic aids and other substances (e.g. growth hormone, Nitric Oxide-containing supplements, creatine-containing supplements, etc.), and a higher frequency of smoking. These results are in agreement with those reported by previous studies [2, 3, 9, 11, 13].

Fifty nine percent of users reported believing they have enough information regarding AAS. However, 50 % of user had no or incorrect information and only 18.2 % of them had appropriate knowledge regarding the potential adverse effect of AAS use. Deficient knowledge about complications of AAS was also reported in previous studies [12]. Interestingly, non-users were as much uninformed as users about the side effects of AAS, even though a significantly larger percentage of them thought they are harmful to health. Supporting the conclusion that spreading awareness about the side effects of AAS may not be the most effective strategy for limiting their abuse. These results show that the attitudes towards AAS do not correspond with knowledge about their harms, and the attitudes towards AAS are more strongly associated with their use than is knowledge of the potential harms.

The reported main source for acquiring AAS was gym coaches (62.1 %), and this is similar to that previously reported by previous studies [1, 10]. Furthermore, it is of concern that even though AAS are illegal in Kuwait, a significant percentage of users (30.8 %) reported being able to acquire AAS from pharmacists and physicians who are knowledgeable regarding the risks of taking AAS. This is all the more significant because oral route of administration was common (which is more hepatotoxic), with 53.2 % administering ≥1 courses of AAS combining ≥ 2 substances; and that is a high usage rate that increases the risk of side-effects. This may present an additional burden to the health service by a relatively young group of adults. From a regulatory perspective, there may be a need to consider tightening the control of distribution of AAS.

The main limitation of this study was that participants were self-selected insofar as 51.5 % (i.e. *n* = 206) of fitness centre attendees failed to return the completed questionnaire. When asked about the reason for not completing the questionnaire virtually all of them replied that they felt it was too long. However, there was no feedback that indicated preferential participation by users compared to non-users or vice versa. Furthermore, the age was given as age brackets, therefore the means and standard deviations could not be calculated. On the other hand, the main strength of this study is that, in the analyses, it factors in the intentions to use AAS in the future at the point when the data was collected. This gives more accurate picture of the relationship between AAS use and other parameters.

## Conclusion

The study concludes that the beliefs and attitudes regarding the effects of AAS on muscle and the harmfulness of AAS are significantly different between users and non-users, this is expected and was already established in the previous studies. However, this study also shows that the presence of intentions to use AAS is associated with attitudes similar to those of AAS users, this suggests that the attitude precedes and anticipates, and therefore is the cause and not the result of, the decision to use AAS.

From our results, it appears that knowledge or ignorance about the potential harms of AAS is not a major factor influencing the use of AAS. The magnitude of concerns about the actual likelihood and the severity of the side effects of AAS are rather far more influential. However, the main factor determining one’s decision to use AAS is the perceived need for AAS to reach one’s goals in the gym. In other words, the factors that leads an individual to use or not use AAS are mainly whether or not that person wants to build bulky muscles, and whether or not he believes AAS are crucial for that purpose. Moreover, peer effect appears to be strikingly important in the misuse of AAS. Given that smoking, as well as the use of multiple substances that are generally considered to be a risk to health, are more frequent amongst AAS users, it is reasonable to suggest that a personality that is associated with addictions or risk taking behaviour may also be associated with AAS use.

The results of this study lead us towards a different conclusion from what was intuitively assumed and usually expected, and from what was recommended in some previous studies. Namely, education regarding the side effects of AAS is not the crucial area to address when effectively decreasing the abuse of AAS is desired. This, however, agrees with the conclusions of some previous publications [5, 6].

## References

[1] Al-falasi, O., Al-dahmani, K., Al-eisaei, K., Al-ameri, S., Al-maskari, F., Nagelkerke, N., & Schneider, J. (2008). Knowledge, attitude and practice of anabolic steroids use among gym users in Al-Ain District, United Arab Emirates, 75–81. http://benthamopen.com/ABSTRACT/TOSMJ-2-75.

[2] Bahrke MS, Yesalis CE, Brower KJ (1998). Anabolic-androgenic steroid abuse and performance-enhancing drugs among adolescents. Child Adolesc Psychiatr Clin N Am.

[3] Baker JS, Graham MR, Davies B (2006). Steroid and prescription medicine abuse in the health and fitness community: a regional study. Eur J Intern Med.

[4] Fitness, C. on S. M. and (1997). Adolescents and Anabolic Steroids: A Subject Review. Pediatrics, 99 (6), 904–908. doi:10.1542/peds.99.6.904. http://pediatrics.aappublications.org/content/99/6/904.full. 9190555

[5] Goldberg L, Bents R, Bosworth E, Trevisan L, Elliot DL (1991). Anabolic steroid education and adolescents: do scare tactics work?. Pediatrics.

[6] Goldberg L, Goldberg L, Elliot D, Clarke GN, MacKinnon DP, Moe E (1996). Effects of a multidimensional anabolic steroid prevention intervention. The Adolescents Training and Learning to Avoid Steroids (ATLAS) Program. JAMA.

[7] Hall RCW, Hall RCW (2005). Abuse of supraphysiologic doses of anabolic steroids. South Med J.

[8] Kious BM (2008). Philosophy on steroids: why the anti-doping position could use a little enhancement. Theor Med Bioeth.

[9] Melia P, Pipe A, Greenberg L (1996). The use of anabolic-androgenic steroids by Canadian students. Clin J Sport Med.

[10] Mohammad H (2014). Anabolic-androgenic steroids amongst Kuwaiti males. Coll Stud J.

[11] Nilsson S, Spak F, Marklund B, Baigi A, Allebeck P (2004). Attitudes and behaviors with regards to androgenic anabolic steroids among male adolescents in a county of Sweden. Subst Use Misuse.

[12] Nojoomi, M., & Behravan, V. (2005). Study of anabolic steroids and the awareness of their complications in bodybuilding athletes in Karaj (2003). RJMS. Retrieved from http://rjms.iums.ac.ir/browse.php?a_code=A-10-1-134&slc_lang=en&sid=1.

[13] Parkinson AB, Evans NA (2006). Anabolic androgenic steroids: a survey of 500 users. Med Sci Sports Exerc.

[14] Santos AM, da Rocha MSP, da Silva MF (2011). Illicit use and abuse of anabolic-androgenic steroids among Brazilian bodybuilders. Subst Use Misuse.

[15] Singhammer J. (2013). Attitudes toward anabolic-androgenic steroids among non-competing athletes in various types of sports – a cross-sectional study –. sport science review, XXII(1–2), 109–128. doi:10.2478/ssr-2013-0006

[16] Nilsson S, Marklund B, Fridlund B (2001). Trends in the misuse of androgenic anabolic steroids among boys 16?17 years old in a primary health care area in Sweden. Scand J Prim Health Care.

[17] Tahtamouni LH, Mustafa NH, Alfaouri AA, Hassan IM, Abdalla MY, Yasin SR (2008). Prevalence and risk factors for anabolic-androgenic steroid abuse among Jordanian collegiate students and athletes. Eur J Public Health.

[18] Thiblin I, Petersson A (2005). Pharmacoepidemiology of anabolic androgenic steroids: a review. Fundam Clin Pharmacol.

[19] Van Amsterdam J, Opperhuizen A, Hartgens F (2010). Adverse health effects of anabolic-androgenic steroids. Regul Toxicol Pharmacol.

[20] Wilson JD (1988). Androgen abuse by athletes. Endocr Rev.

